# Removal and analysis of foodborne-associated pathogens via peptide functionalized nanoparticles

**DOI:** 10.1007/s00253-026-13922-x

**Published:** 2026-07-06

**Authors:** Emily Hausen, Sebastian Knorr, Stefan Lyer, Christoph Alexiou, Rainer Tietze, Sonja Lick

**Affiliations:** 1 Department of Otorhinolaryngology, Head and Neck Surgery, Section of Experimental Oncology and Nanomedicine (SEON), Else Kröner-Fresenius Stiftung Professorship, Uniklinikum Erlangen, Erlangen, Germany; 2 Department of Otorhinolaryngology, Head and Neck Surgery, Section of Experimental Oncology and Nanomedicine (SEON), Professorship for AI assisted Nanomaterials, Uniklinikum Erlangen, Erlangen, Germany; 3 Department of Safety and Quality of Meat, Max Rubner-Institute (MRI), Kulmbach, Germany

**Keywords:** SPION, GP-340, *L. monocytogenes*, Pathogen, Foodborne illness, Pathogen detection

## Abstract

**Abstract:**

Foodborne illnesses still remain a major health issue, as the WHO reported 600 million diseases and 420,000 deaths worldwide in 2010. Most of them were caused by pathogens like *Campylobacter* spp., *Salmonella enterica*, *Escherichia coli*, and *Listeria monocytogenes*. Due to the short shelf life of fresh meat and dairy products, a rapid detection of pathogens in contaminated food products is essential. Since reference methods for microbial food analysis require time-consuming nonselective and selective culturing procedures, the need for more efficient approaches is given. In this study, we employ superparamagnetic iron oxide nanoparticles (SPIONs) functionalized with the peptide KRQGRVEVLYRASWGTV derived from the salivary protein GP-340, to successfully remove different species of food-related microorganisms (*Pseudomonas paracarnis*, *Campylobacter jejuni*, *Escherichia coli*, *Enterococcus faecalis*, *Salmonella enterica*, and *Listeria monocytogenes*) from pure media and homogenates derived from retail meat products. We discovered differences in extraction efficiencies from pure culture ranging from 18% (*C. jejuni*) to complete extraction (*E. faecalis*, *L. monocytogenes* and *P. paracarnis*) depending on the organism. It was also shown that *L. monocytogenes* could successfully be removed from co-culture with a high surplus of non-pathogenic *P. paracarnis*, and also from food matrices.

**Key points:**

• *SPION*^*Cit*^*can be functionalized with a GP-340 derived peptide (SPION*^*Pep*^*)*

• *SPION*^*Pep*^* can immobilize and remove L. monocytogenes from pure and co-culture with high surplus of non-pathogenic P. paracarnis*

• *Removal of L. monocytogenes is also possible in complex food matrices*

**Supplementary Information:**

The online version contains supplementary material available at 10.1007/s00253-026-13922-x.

## Introduction

Foodborne illnesses remain a major health issue. The WHO reported 600 million diseases and 420,000 deaths worldwide (2010), caused by 31 foodborne hazards, most of them due to pathogens like *Campylobacter* spp., *Salmonella enterica*, and *Escherichia coli* (Havelaar et al. 2015). *Listeria monocytogenes* also can cause severe foodborne diseases, called human listeriosis, manifesting from subclinical gastro-enteritis to invasive diseases, which are categorized in three forms: (i) pregnancy-associated and neonatal listeriosis, (ii) bacteriemia or septicemic listeriosis and (iii) central nervous system (CNS) infection (Koopmans et al. 2023). It is essential to maintain food quality and consumer safety by rapid detection of pathogens in contaminated food, especially with short shelf live products like fresh meat and dairy. Therefore, timely detection of pathogens is important, but traditional culturing methods involve nonselective and selective enrichment with subsequent confirmation of the illness causing microbe. To meet the demand for rapid detection, high-performing approaches employ a range of methods, including immunological, molecular, and sequencing techniques, as well as matrix-assisted laser desorption ionization-time-of-flight mass spectrometry (MALDI-TOF) and spectroscopic methods (Ferone et al. 2020).

Superparamagnetic iron oxide nanoparticles (SPIONs) are used for a variety of applications like magnetic drug targeting (Matuszak et al. 2018), hyperthermia (Pucci et al. 2022), for magnetic particle spectroscopy (Friedrich et al. 2023b; Vogel et al. 2022), and loading of T cells (Boosz et al. 2021; Mühlberger et al. 2019). Prior discoveries also used peptide functionalized SPIONs to address pathogens for diagnostic purposes in bloodstream infections (Friedrich et al., 2022a; Friedrich et al., 2022; Friedrich et al. 2023a). Diagnosing the sepsis causing pathogen usually requires culturing steps and therefore time, but is essential for the right treatment (Kalil et al. 2017; Ransom et al. 2021). The particles used in these studies showed successful extraction of lipopolysaccharides (LPS), lipoteichoic acid (LTA) and pathogens from media and blood. When coupled to additional detection-methods like critical offset magnetic particle spectroscopy (COMPASS) (Friedrich et al. 2023b), this technique enables faster sample preparations and analysis compared to standard cultivation methods while maintaining sensitivity (Friedrich et al., 2022b).

Said peptide derives from the native saliva protein GP-340, also known as Deleted in Malignant Brain Tumors 1 (DMBT1), which is renowned for its bacteria binding properties. These characteristics are attributed to the SRCR (scavenger receptor cysteine-rich) domains and mainly the binding motif VEVLXXXXW (Bikker et al. 2017, 2004). Based on this knowledge, we propose the idea of similar applications in relation to identifying pathogens for food safety.

This study uses the peptide KRQGRVEVLYRASWGTV bound on the surface of citrate coated SPIONs to magnetically remove the food-related microorganisms *Pseudomonas paracarnis*, *Campylobacter jejuni*, *Escherichia coli*, *Enterococcus faecalis*, *Salmonella enterica*, and *Listeria monocytogenes* in pure and co-culture, as well as in the food matrices meat spread and minced meat.

## Materials and methods

### Materials

Iron(II) chloride tetrahydrate, iron(III) chloride hexahydrate and 2-(N-Morpholino)ethanesulfonic acid hydrate (MES hydrate) were purchased by Merck KGaA (Darmstadt, Germany), Ringer’s solution from Fresenius Kabi (Bad Homburg, Germany), 1-Ethyl-3-(3-dimethylaminopropyl)carbodiimide (EDC) and N-Hydroxysulfosuccinimide Sodium Salt (S-NHS) from TCI Deutschland GmbH (Eschborn, Germany). Trisodium citrate dihydrate, acetone, ammonia (NH_3_), boric acid, sodium chloride (NaCl), trifluoroacetic acid (TFA), acetonitrile (ACN) and nitric acid (HNO_3_) were obtained by Carl Roth GmbH + Co. KG (Karlsruhe, Germany). The peptide KRQGRVEVLYRASWGTV was purchased from ProteoGenix (Schiltigheim, France). The water used for the experiments came from a MilliQ Purification System (Merck KGaA, Darmstadt, Germany). All materials were used without further purification.

### Bacterial strains and general culture conditions

*Pseudomonas paracarnis* DSM 111363^T^, *Campylobacter jejuni* DSM 4688, and *Escherichia coli DSM 1103* were derived from Leibniz-Institut DSMZ-Deutsche Sammlung von Mikroorganismen und Zellkulturen (Braunschweig, Germany). *Enterococcus faecalis* and* L. monocytogenes* serovar 4b are part of the institutional strain deposit of the Max Rubner-Institute (Kulmbach, Germany). *Salmonella enterica* subsp. *enterica* ser. *derby* (*S. derby*) was provided by the Friedrich-Loeffler-Institute (Mariensee, Germany). For all strains, cryobank stocks were created and stored at − 80 °C. *C. jejuni* was grown in Bolton broth (Oxoid, Basingstoke, United Kingdom) for liquid cultures or on modified Charcoal Cefoperazone Deoxycholate Agar (mCCDA; VWR International GmbH, Darmstadt, Germany) plates (solidified with 1.5% agar) at 42 °C under microaerobic conditions (82.5% N_2_, 10% H_2_ and 7.5% CO_2_) for 48 h for colony formation. The atmosphere was established in an anaerobic jar using a Whitley Jar Gassing System (Don Whitley Scientific Ltd., Bingley, United Kingdom). Unless stated otherwise, *P. paracarnis*, *L. monocytogenes* serovar 4b, *E. coli*, *E. faecalis*, and *S. derby* were grown in Standard I nutrient Broth (Merck KGaA, Darmstadt, Germany) and plated on Plate Count Agar (PCA) (1.2%) (Carl Roth GmbH + Co. KG, Karlsruhe, Germany) incubated under aerobic conditions at 30 °C (*P. paracarnis*) or 37 °C (other strains).

### Synthesis of SPION^*Cit*^

SPION^Cit^-particles were synthesized using a modified protocol of the coprecipitation method (Mühlberger et al. 2019). In short 745.5 mg iron (II) chloride tetrahydrate and 2027.5 mg iron (III) chloride hexahydrate (molar ratio of 1:2) were dissolved in 50 ml MilliQ water. They were put under argon atmosphere and stirred at room temperature at 400 rpm. After 10 min 6.25 ml 25% NH_3_ were added and stirred for another 10 min. Subsequently 15 ml of a 1 M citrate solution was injected and simultaneously heated to 90 °C for 30 min. After cooling with an ice bath, the particles synthesized were washed magnetically five times with acetone and stored in MilliQ water at 8 °C until further use.

### Peptide Functionalization SPION^*Cit*^

**Fig. 1:**
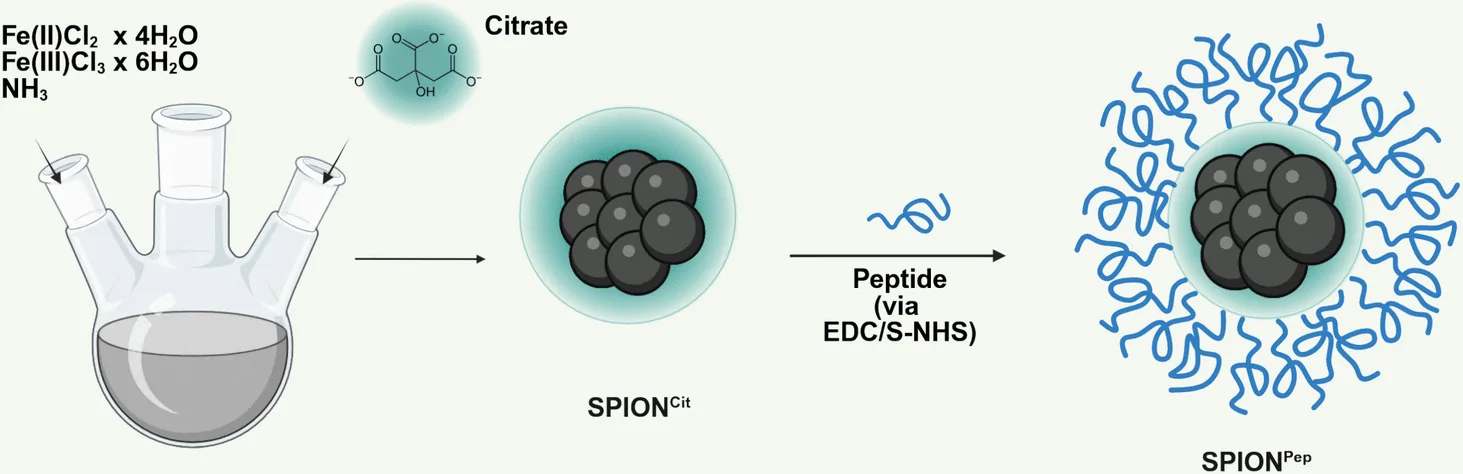
Synthesis of SPION^Cit^ and subsequent binding of peptide on the particle surface. In the first step, magnetic nanoparticles are obtained by coprecipitation of iron (II) and iron (III) chloride in basic milieu. Then peptide KRQGRVEVLYRASWGTV is bound via EDC/S-NHS-chemistry. Created with BioRender.com

A modified protocol by Thermo Fisher Scientific (2020) was used to bind peptide KRQGRVEVLYRASWGTV via terminal amino groups and EDC/S-NHS chemistry on SPION^Cit^. The citrate coated magnetic nanoparticles were put in a 100 mM MES buffer at pH 5.5 in a 1:1 ratio to achieve an iron content of 5 mg/ml. EDC and S-NHS dissolved in 50 mM MES buffer pH 5.5 were added, vortexed and put in an overhead rotator for 15 min to activate the carboxy groups. In this time the peptide was dissolved in a 0.5 M HEPPS buffer pH 8.5 (2 µmol/ml peptide) using an ultrasonic bath (SONOREX DIGITEC DT, BANDELIN electronic GmbH & Co. KG, Berlin, Germany). The solution was added and the mixture was left on the overhead rotator for 1 h. To quench the reaction 1 M borate buffer pH 9 was added; the quenching time was 30 min. The SPIONs functionalized with peptide were washed several times magnetically with MilliQ water and saturated NaCl solution. The supernatants from these washing steps were kept at 8 °C and residual peptide was quantified via high pressure liquid chromatography (HPLC). Resulting SPION^Pep^ were stored in H_2_O at 8 °C until further use. A schematic overview of synthesis and subsequent peptide binding is visualized in Fig. 1.

### Characterization

The resulting SPIONs were characterized based on their iron content using a microwave plasma atomic emission spectroscopy (MP-AES; MP-4200; Agilent Technologies, Santa Clara, California, USA) and a commercially available iron standard solution (AnalytiChem GmbH, Duisburg, Germany). Diluted nanoparticles were dissolved in nitric acid 65% at 95 °C for 10 min and the resulting solution was then filled up to 2 ml with MilliQ water. Each measurement was performed in technical triplicates. Further characterization was then done at desired concentrations.

The particles were then characterized by size using a Zetasizer Nano ZS (Malvern Panalytical, Almelo, The Netherlands). SPION^Cit^ and SPION^Pep^ were diluted in MilliQ water to the desired concentration of 50 µg iron/ml and then analyzed at 25 °C (refractive index 1.33, viscosity 0.8872 mPA s, backscattering mode at 173°). Each sample was analyzed in triplicates.

The remaining amount of peptide after functionalization was determined by high pressure liquid chromatography (HPLC; KNAUER Wissenschaftliche Geräte GmbH, Berlin, Germany) using a reversed phase C18 column (Eurosil Bioselect 300–5; KNAUER Wissenschaftliche Geräte GmbH, Berlin, Germany) with an UV/vis detector (KNAUER Wissenschaftliche Geräte GmbH, Berlin, Germany) after centrifuging the collected supernatants for 5 min at 14,100 rcf to ensure the absence of particles. The analysis gradient started with 80% H_2_O with 0.1% TFA and 20% ACN with 0.1% TFA, ending at 50% each over a time of 15 min at 30 °C thermostat (KNAUER Wissenschaftliche Geräte GmbH, Berlin, Germany) temperature. The detector wavelengths were set to 220 and 280 nm.

### Magnetic extraction of different bacteria from Ringer’s solution

The bacterial strains used in this separation assay were *P. paracarnis* as an ubiquitarian non-pathogenic bacterium, *L. monocytogenes*, *C. jejuni*, *E. coli*, *E. faecalis* and *S. derby*.

Overnight cultures of the strains were prepared in liquid medium and OD_600_ was measured with a Photometer DR 3900 (Hach Lange GmbH, Berlin, Germany) following dilution of the cultures to an OD_600_ of 0.2. Assuming approximately ~ 10^8^ cells/ml for an OD of 0.2, 10 × serial dilutions into pure Ringer’s solution were prepared until reaching the indicated cell concentrations.

The extraction process was based on an already established process by Friedrich et al. (2022b). Nine hundred microliters of the bacterial solution and 100 µl of SPION^Pep^ particle suspension with a concentration of around 3–3.5 mg Fe/ml (final concentration of nanoparticles in the sample 300–350 µg Fe/ml) were put in a 2 ml tube (Eppendorf SE, Hamburg, Germany) and mixed in an overhead rotator for 10 min at 60 rpm. Subsequently, the tubes were put in a magnetic tray for 3 min. The resulting supernatant was carefully harvested and used for further analysis to detect bacterial leftovers after separation. Removed magnetic particles were washed by redispersing them in 1000 µl Ringer’s solution and carefully pipetting them. Then the SPIONs were again separated for 3 min in the magnetic tray. This second supernatant was discarded and the particles were dispersed in 500 µl Ringer’s solution for plating. Each strain and concentration were analyzed in triplicates.

As reference (control groups) for total bacterial counts prior to separation 900 µl bacteria solution and 100 µl sterile H_2_O (bidest.) were incubated the same as previously described without magnetic separation and analyzed via quantitative plate counting.

### Quantitative extraction of bacterial pure- and co-cultures

Samples containing each approximately 10^3^ or 10^4^ cells/ml of *P. paracarnis*, *L. monocytogenes*, *C. jejuni*, *E. coli*, *E. faecalis*, or *S. derby* in Ringer’s solution underwent the extraction procedure for bacteria by SPIONs. To determine the ability to extract different bacteria in pure culture functionalized SPION^Pep^ were tested. After the extraction 100 µl of the nanoparticle fractions containing extracted bacteria (nanoparticle fraction), the supernatants containing non-extracted bacteria (supernatant fraction) and a non-treated control fraction were plated in logarithmic mode with a spiral plater Whitley Wasp Touch (Don Whitley Scientific Limited, Bingley, United Kingdom). MCCDA (*C. jejuni*) and PCA (all other strains) plates were incubated as described under general culture conditions and the absolute number of grown colonies per ml was determined with an automated aCOLyte 3 HD colony counting device (Synoptics, Cambridge, United Kingdom).

Extraction efficiencies were calculated as the ratio of colony forming units per ml (CFU/ml) recovered in the particle fraction to CFU/ml of the untreated control, expressed as percentage:

$$E= \frac{\overline{{\mathrm{C}\mathrm{F}\mathrm{U} }_{\mathrm{p}\mathrm{a}\mathrm{r}\mathrm{t}\mathrm{i}\mathrm{c}\mathrm{l}\mathrm{e}}}}{\overline{{\mathrm{C}\mathrm{F}\mathrm{U} }_{\mathrm{c}\mathrm{o}\mathrm{n}\mathrm{t}\mathrm{r}\mathrm{o}\mathrm{l}}}} \times 100$$

with *E* being the efficiency and $\overline{{\mathrm{C}\mathrm{F}\mathrm{U} }_{\mathrm{p}\mathrm{a}\mathrm{r}\mathrm{t}\mathrm{i}\mathrm{c}\mathrm{l}\mathrm{e}}} \mathrm{a}\mathrm{n}\mathrm{d} \overline{{\mathrm{C}\mathrm{F}\mathrm{U} }_{\mathrm{c}\mathrm{o}\mathrm{n}\mathrm{t}\mathrm{r}\mathrm{o}\mathrm{l}}}$ representing the mean CFU/ml of the particle fraction and the untreated control, respectively.

To validate the extraction procedure, the sum of CFU/ml recovered in the particle and supernatant fractions was compared to the untreated control using the Mann–Whitney *U* test with False Discovery Rate (FDR) correction (two-stage step-up method of Benjamini, Krieger and Yekutieli; GraphPad Prism 10.2.0).

To confirm quantitative extraction of *L. monocytogenes* in the presence of other ubiquitarian bacteria of the meat microbiota, three co-cultures with a surplus of *P. paracarnis* were prepared: 10, 100, and 1000 *L. monocytogenes* cells per ml with 10^6^ per ml *P. paracarnis* each. The extraction process was handled the same way as in pure culture. For quantitative enumeration of living *L. monocytogenes* in the three fractions (non-treated control, nanoparticles, supernatant), the whole sample volume (1 ml for non-treated control and supernatant, 0.5 ml for nanoparticles) of each fraction was directly plated on Chromocult *L.* selective Agar acc. OTTAVIANI and AGOSTI (Merck KGaA, Darmstadt, Germany), allowing only *L. monocytogenes* to grow. After incubating the agar plates for 24 h at 37 °C under aerobic conditions, absolute counts for *L. monocytogenes* stated as CFU/ml were determined for the samples using an aCOLyte 3 HD colony counting device (Synoptics, Cambridge, United Kingdom). Extraction efficiencies were calculated the same way as stated before.

### Quantitative extraction of *L. monocytogenes* from meat matrices

**Fig. 2:**
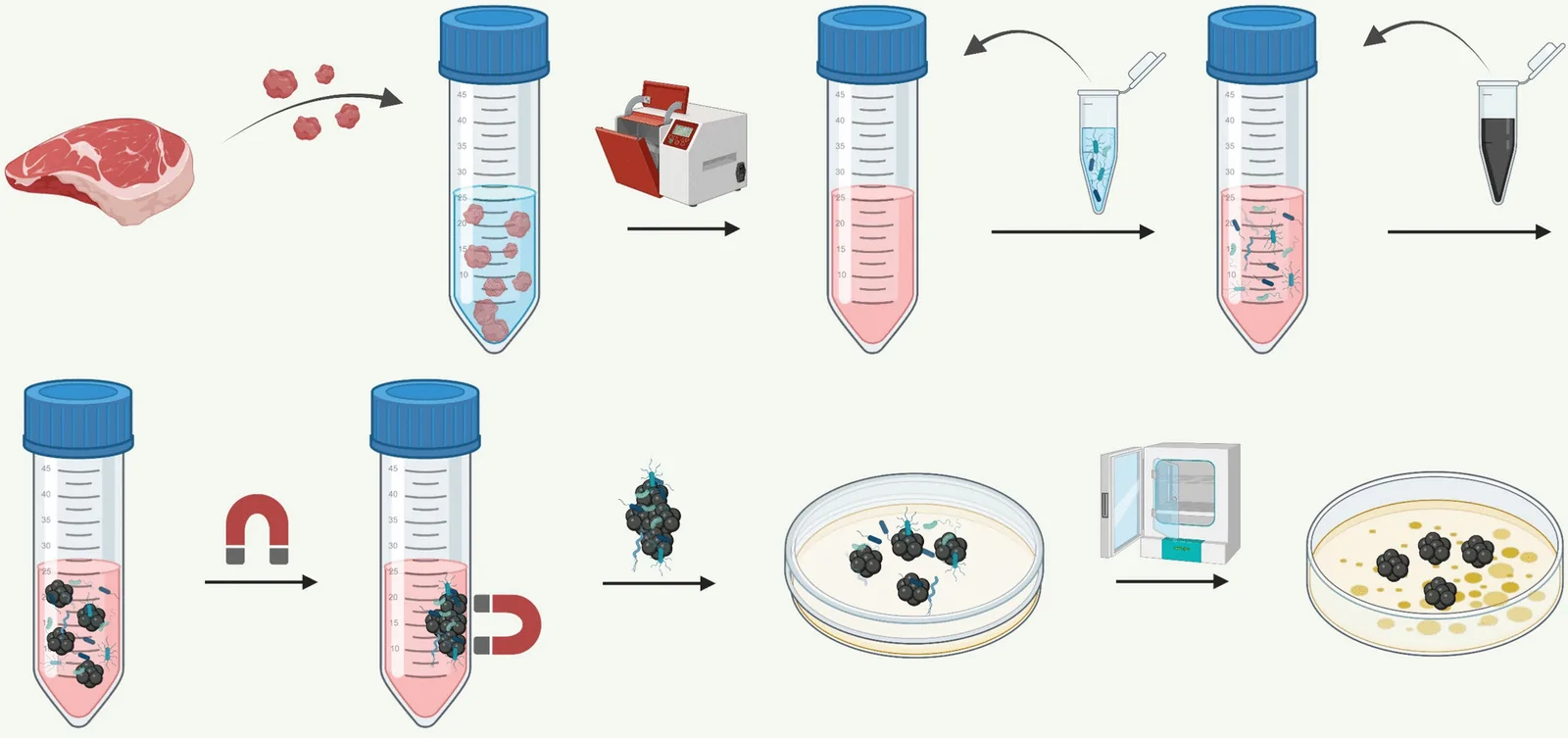
Schematic separation of pathogens from food matrices. In short, a food sample is prepared in the stomacher then spiked with a certain bacteria load. Magnetic nanoparticles are added and after incubation the SPION^Pep^ are magnetically extracted and plated. Bound pathogens will grow on (selective) plates after incubation. Created with BioRender.com

Fresh retail products of minced meat and smoked sausage spread were purchased from local supermarkets in Bavaria, Germany. As a meat-based matrix for bacterial SPION^Pep^ extraction, retail meat products were prepared based on the International Standard DIN EN ISO 6887–2:2017 (International Organization for Standardization, 2017). Shortly, 20 g of each meat product were aseptically removed with an ethanol-sterilized spatula and placed into a stomacher bag (VWR Avantor, Radnor, Pennsylvania). 180 ml of sterile Ringer’s solution was mixed in with a Stomacher 400 Circulator (Seward Ltd, Worthing, United Kingdom) for 2 min at 230 rpm. Fifty to 100 ml of the meat suspension was withdrawn from the bag and used for further analysis. 1 ml of *L. monocytogenes* from an overnight culture (OD_600_ = 0.2 corresponding to approximately ~ 10^8^ cells/ml) was spiked into the meat suspension (9 ml) and diluted further to reach indicated cell concentrations. This resulted in 4 different meat matrix samples containing approximately 10, 10^2^, 10^3^, 10^4^ cells/ml meat suspension, respectively. The non-spiked meat suspension was used as *L. monocytogenes*-free control. To 900 µl of the prepared meat suspensions spiked with *Listeria* (or *Listeria*-free) 100 µl SPION^Pep^ were added and incubated for 10 min on the overhead rotator. After 3 min of magnetic separation, the supernatant was kept on ice for plating on Chromocult *L.* selective Agar acc. OTTAVIANI and AGOSTI (Merck KGaA, Darmstadt, Germany). The particles were washed with 1000 µl Ringer’s solution and again magnetically separated. The washed SPION^Pep^ were then dispersed in 500 µl Ringer’s solution and plated. Each spiked meat suspension dilution was tested in triplicates. 100 µl of the 10^4^ (logarithmic mode), 10^2^ and 10^3^ (linear mode) cells/ml samples as well as 1 ml of the 10 cells/ml samples were plated using the Spiral Plater Whitley Wasp Touch (Don Whitley Scientific Limited, Bingley, United Kingdom). Absolute CFU/ml counts were determined after incubation for 24 h at 37 °C under aerobic conditions and efficiencies calculated as stated before. A schematic overview of the extraction process from meat matrices is visualized in Fig. 2.

### 3D confocal laser scanning microscopy

A Leica TCS SP8 X CLSM (Leica Microsystems, Wetzlar, Germany) equipped with an argon laser, PMT and HyD detector, and HC PL APO 63X/1.20 W CORR CS2 lens was used for detecting SPION^Pep^-nanoparticles as well as fluorescent signals of stained bacteria. To enable 3D-imaging of nanoparticle-bacteria complexes, samples containing *P. paracarnis* extracted with SPION^Pep^ were embedded in a 3D-imaging-matrix consisting of 1.5% (w/v) NEEO ultra quality agarose dissolved in Ringer’s solution. Imaging of nanoparticles was performed using a 494-nm excitation laser wavelength with detecting the reflected light from 495–549 nm. *P. paracarnis* cells were stained with 100 µM SynaptoRed™ C2 (Equivalent to FM®4–64, Biotium). Fluorescent signals were excited with the laser line at 494 nm and emission was detected from 644 to 735 nm. For 3D-imaging a z-step size of 182 nm, a resolution of 192 × 192 × 36 px with 4 times frame averaging per picture and zoom factor of 21 × was chosen via the “Lightning” software module of the LASX (Leica Microsystems, Wetzlar, Germany). LASX was furthermore used to generate displayed images and quantify reflected light signals.

## Results

### Synthesis of SPION^*Cit*^ and subsequent peptide functionalization

For this study, SPION^Cit^ were synthesized using alkaline coprecipitation and have been further functionalized with peptide KRQGRVEVLYRASWGTV, as a derivate of the saliva protein GP-340, (SPION^Pep^).

Used SPION^Cit^ had a hydrodynamic diameter (z-average) of 56 ± 7 nm with a PDI of 0.219 ± 0.008. Following binding, the particles grew and became polydisperse. This forming of bigger clusters showed in an increased z-average of 500 ± 66 nm with a PDI of 0.728 ± 0.167 (see supplementary Fig. A1). However, *z*-average is not necessarily valid for SPION^Pep^ as multiple peaks occur in the intensity rated size distributions (Bhattacharjee 2016).

To determine whether the functionalization was successful, the supernatants after the reaction and washing the particles were analyzed via HPLC–UV. This way the absence of peptide in the supernatant directly after binding was shown (see supplementary Fig. A2) and indicate binding on SPION^Cit^. Furthermore, it does not wash off from the magnetic nanoparticles as no peptide peak can be found after washing the particles.

### Extraction of pathogens from pure and co-culture

Previous studies have already proven the successful recovery of pathogens from different media, e.g., blood (Friedrich et al. 2022b). One of this study´s main focus was to determine the extraction efficiency of pathogenic and spoilage bacteria from pure and co-culture, but also from food media.

**Fig. 3:**
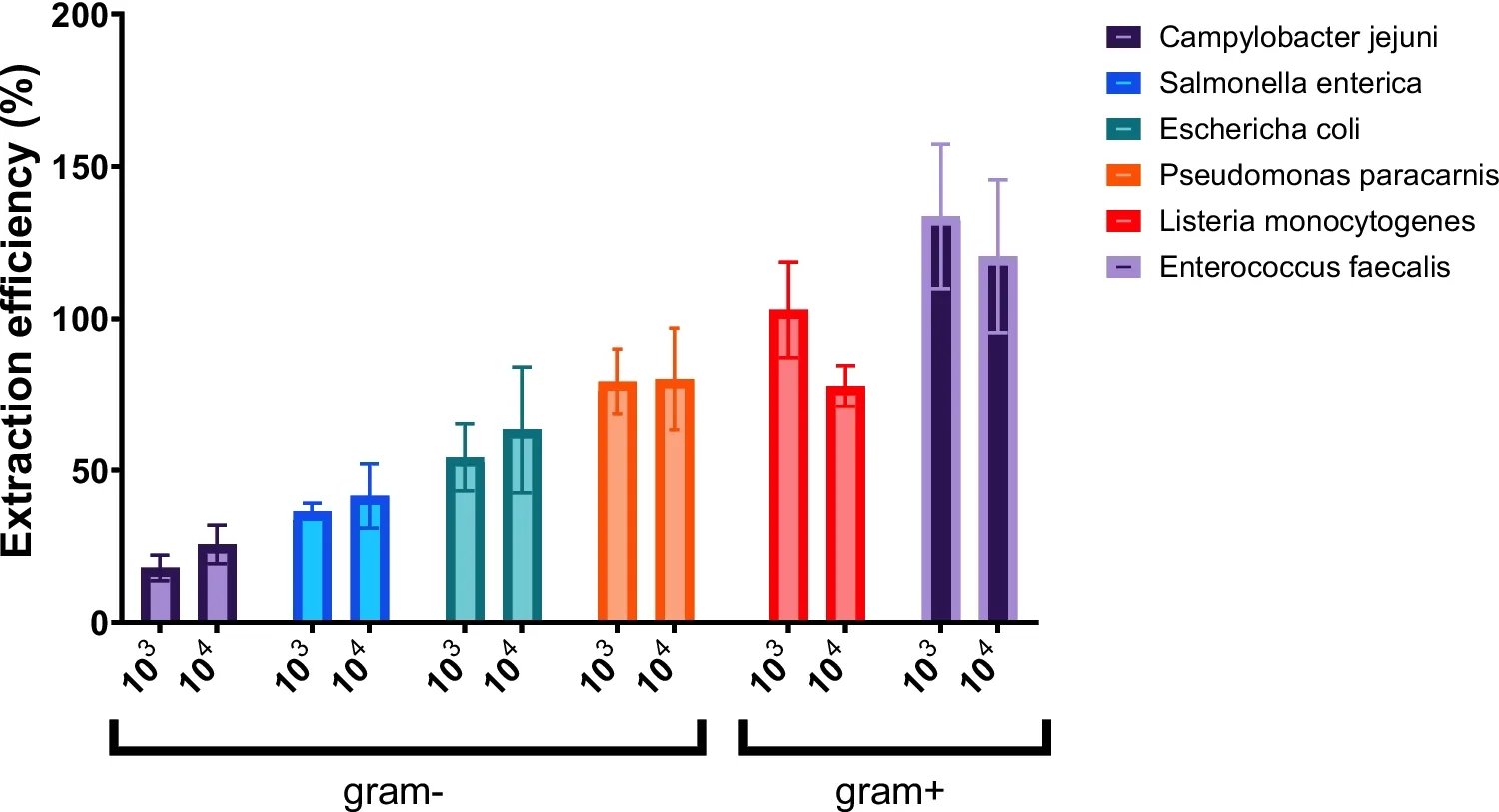
Pure culture extraction efficiencies of different bacteria at 10^3^ and 10^4^ CFU/ml, sorted by gram staining. Tested gram-negative bacteria are *C. jejuni*, *S. enterica*, *E. coli* and *P. paracarnis*; gram-positive are *L. monocytogenes* and *E. faecalis*

Pure culture extraction efficiencies with SPION^Pep^ are displayed in Fig. 3, showing differences in the separation of bacteria. While *C. jejuni* showed the lowest extraction efficiency of tested bacteria (18% at 10^3^, 26% at 10^4^ CFU/ml), while the separation efficiency of *E. faecalis* (134% at 10^3^, 121% at 10^4^ CFU/ml) and *L. monocytogenes* (103% at 10^3^, 78% at 10^4^ CFU/ml) was maximal. We were able to see differences in extraction efficiencies based on gram-status. Here, gram-positive microorganisms show higher efficiencies than gram-negative bacteria with the exception of *P. paracarnis* (80% at 10^3^ and 10^4^ CFU/ml). Other gram-negative bacteria like *S. enterica* (37% at 10^3^, 42% at 10^4^ CFU/ml) and *E. coli* (54% at 10^3^, 63% at 10^4^ CFU/ml) were able to be extracted from pure culture. Raw CFU/ml data for the extraction with SPION^Pep^ are shown in supplementary Table A1. Furthermore, no significant difference was detected between the control and the combined particle and supernatant fractions for any tested species (Mann–Whitney *U* test, all q > 0.05), confirming the validity of the extraction procedure (see supplementary Fig. A3). Unmodified SPION^Cit^ could not be employed as a control due to their high colloidal stability in Ringer’s solution, which prevents efficient magnetic separation (see supplementary Fig. A4).

**Fig. 4:**
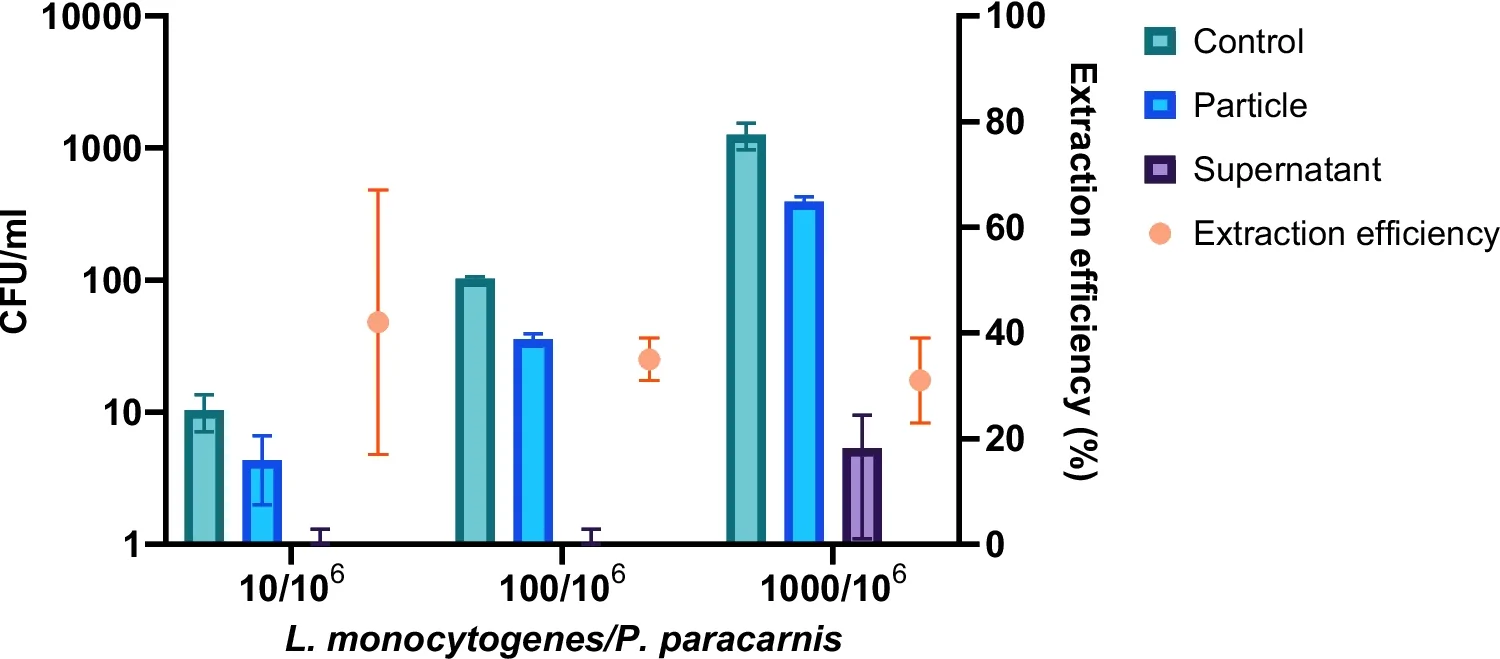
Extraction efficiency of *L. monocytogenes* in co-culture with *P. paracarnis* as an ubiquitarian non-pathogenic microorganism. Extraction efficiencies were 42% for 10/10^6^ CFU/ml, 35% for 100/10^6^ CFU/ml and 31% for 1000/10^6^ CFU/ml. Shown are the control, particle and supernatant groups and the extraction efficiency of each concentration

Pathogens like *L. monocytogenes* are usually outnumbered on food products by members of the general spoilage microbiota. Thus, it is important to verify the recovery rate of less abundant species in the presence of a high bacterial background (e.g., *P. paracarnis* as a representative of spoilage flora). This was tested with *L. monocytogenes*, as it also has a good extraction efficiency in pure culture (Fig. 4). Tested concentrations were 10, 100, and 1000 CFU of *L. monocytogenes* in the presence of 10^6^ *P. paracarnis*. Separation efficiencies were 42% for 10/10^6^, 35% for 100/10^6^, and 31% for 1000/10^6^. Raw CFU/ml data is presented in supplementary Table A2.

### Extraction of pathogens from food matrices

**Fig. 5:**
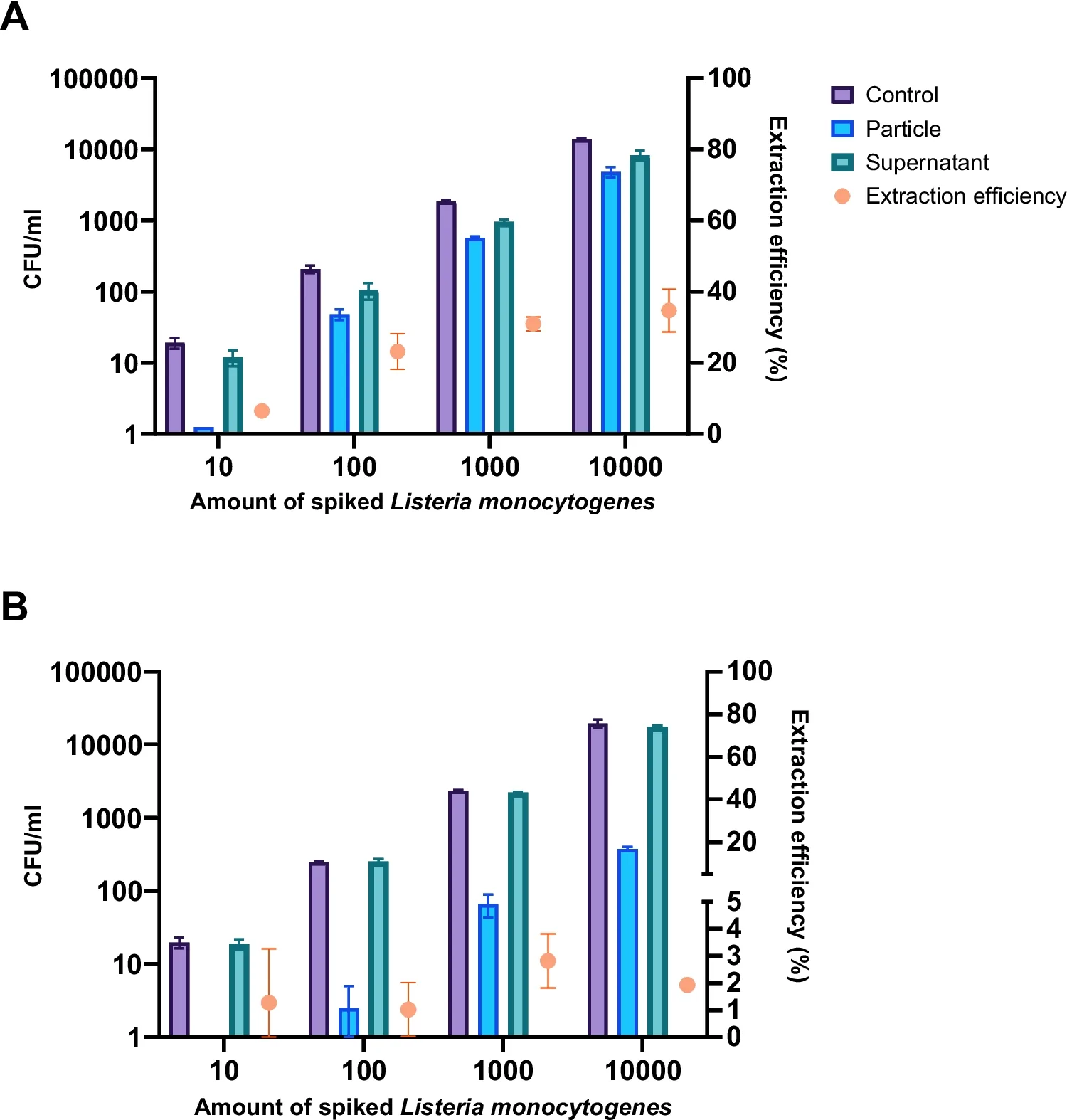
Extraction efficiencies of *L. monocytogenes* in meat matrices, meat spread (A) and minced meat (B). *L. monocytogenes* was spiked to a prepared food matrix to total concentrations of 10, 100, 1000 and 10000 CFU/ml each. A) Extraction efficiencies were 7% for 10, 23% for 100, 31% for 1000 and 35% for 10000 CFU/ml *L. monocytogenes* in meat spread. B) The extraction efficiencies of *L. monocytogenes* from minced meat were: 1% for both 10 and 100 CFU/ml, 3% for 1000 and 2% for 10000 CFU/ml

To test the extraction efficiency under the presence of food matrix particles, two different meat-based matrices were chosen for spiking experiments: meat spread and minced meat. Prepared meat suspensions were tested *L. monocytogenes*-free and spiked with 10, 100, 1000, and 10,000 CFU/ml *L. monocytogenes*. In Fig. 5, the difference in extraction efficiencies of the said pathogen between tested food-matrices is shown. Raw CFU/ml data for extractions in both meat spread and minced meat are shown in supplementary Table A3.

For meat spread, the highest separation frequency was found at 10,000 CFU/ml (35%). Fewer concentrations of *L. monocytogenes* showed decreasing extraction efficiencies: 31% at 1000, 23% at 100, and 7% at 10 CFU/ml.

Even less efficient separations of *L. monocytogenes* were seen in the minced meat matrix: 1% for both 10 and 100 CFU/ml, 3% for 1000, and 2% for 10,000 CFU/ml.

### 3D confocal laser scanning microscopy

**Fig. 6:**
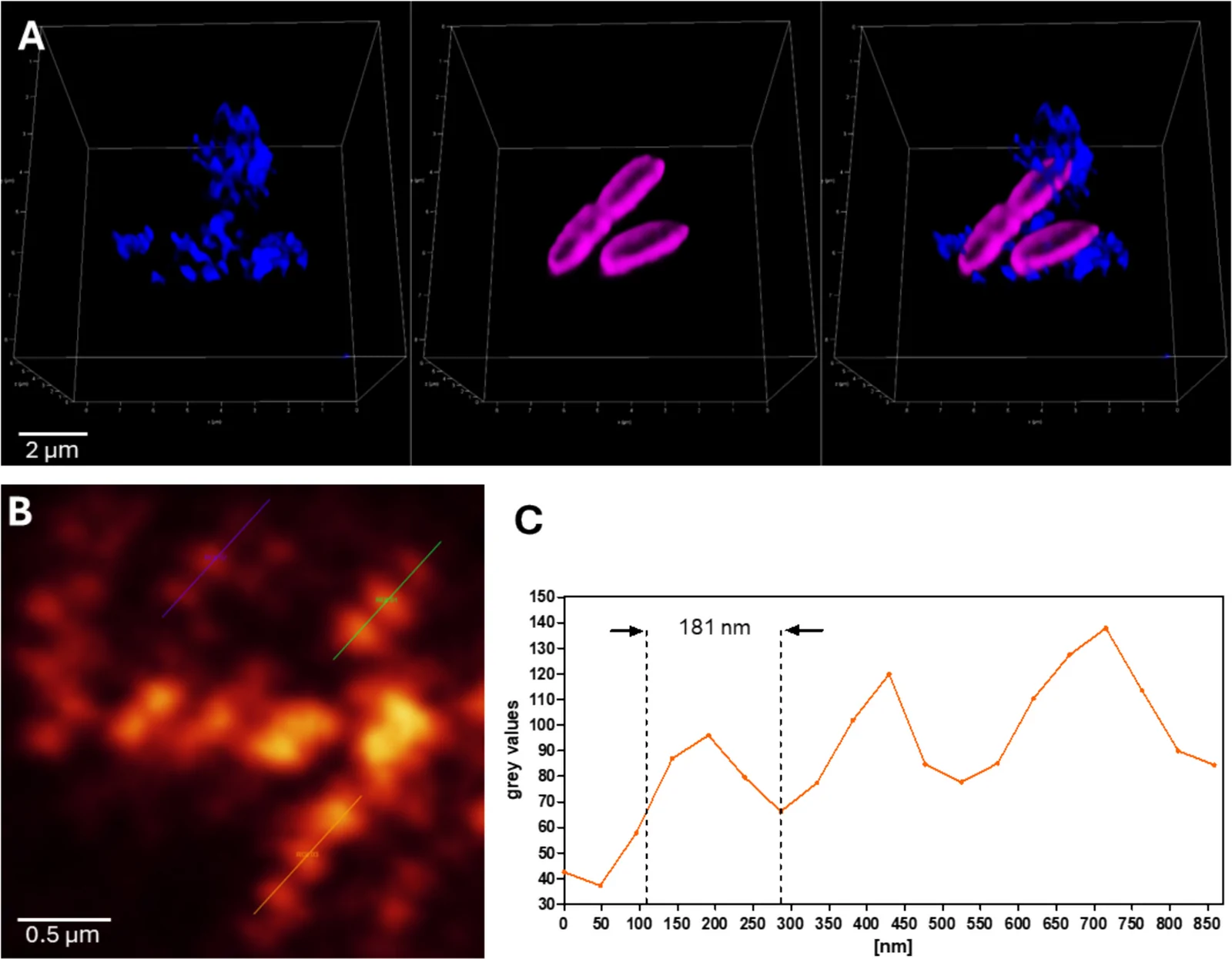
Confocal laser scanning microscopy (CLSM) image of SPION^Pep^-labelled *Pseudomonas* cells. 3D-scan of a *Pseudomonas*-SPION^Pep^ complex in reflectance mode visualizing SPION^Pep^ (left panel), fluorescence labeled *Pseudomonas* cells (middle panel, labelled with FM4-64 analogue SYNAPTO-Red) and overlay (right panel). B) Magnified high-resolution single section visualization of SPION^Pep^ nanoparticles with three regions of interest (ROI) drawn through lined up nanoparticles. C) Related intensity profile to ROI 3 (orange) of the sectioning (B) with black dotted lines indicating the full width half maximum of a single nanoparticle

We used labelling free confocal reflectance microscopy (CRM) in combination with fluorescence-stained *P. paracarnis* to visualize the SPION^Pep^-bacteria complex. CRM has been previously shown to be suitable to distinguish anorganic nanoparticles from organic cells (Friedrich et al. 2022c). *P. paracarnis* cells were pre-stained with a FM4-64 equivalent that incorporates into the bacterial membrane, thus allowing imaging of the outer shape of bacteria. After applying the nanoparticle extraction protocol, nanoparticle-bound bacteria were imbedded into a 3D-matrix to allow stable z-sectional imaging. Imaging of multiple z-stacks confirmed that nanoparticles were concentrated around single *P. paracarnis* cells (Fig. 6A). In less dense particle clusters high-resolution images revealed additional details of single nanoparticles (Fig. 6B). An intensity profile measurement of single particles shows a full width half maximum of about 181 nm.

## Discussion

Superparamagnetic Iron Oxide Nanoparticles were successfully functionalized with the salivary protein GP-340 derived peptide, allowing separation of pathogens, e.g., *L. monocytogenes*, from pure as well as co-culture, but also from food matrices. In Fig. 3, it can be seen that *E. faecalis* shows extraction efficiencies over 100%. *E. faecalis* possesses a coccoid cell shape and usually occurs in two or more cells per chain or cluster (Schleifer & Kilpper-Bälz 1984), which might easily brake during the addition of particle suspension and handling the samples. Thus, slight differences in the growth behavior between particle and control fraction might be responsible for this result. Since standard deviations were rather high with 25 and 24% and supernatants were free of remaining *E. faecalis*, we conclude that the recovery rate for this bacterium must have been almost 100%.

It has been demonstrated that gram-positive bacteria have slightly higher extraction efficiencies than gram-negative ones, with the exception of *P. paracarnis*, which was also able to be separated almost entirely. Former studies also showed the successful separation in Ringer’s solution of gram-positive *Staphylococcus aureus* and *Streptococcus pneumoniae* and even lipoteichoic acid alone (Friedrich et al., 2022a). They were also able to extract different types of lipopolysaccharides (LPS), an essential in the gram-negative cell membrane (Friedrich et al., 2022b).

The gram-negative *C. jejuni*, as the most common cause of foodborne diseases, could not be removed efficiently from pure culture. (26% at 10^4^ and 18% at 10^3^ CFU/ml). It is a helical-shaped pathogen, with the ability to move and form viable but noncultural stages (VBNC) under certain conditions (Chaveerach et al. 2003). It also has unique lipooligosaccharides (LOS) in its cell membrane causing autoimmune responses because of their similarity to human gangliosides. As its LOS is lacking the O-antigen characteristics that are found in other gram-negative species (Naito et al. 2010), this could be further investigated as a possible explanation for the insufficient binding to the SPION^Pep^-particles and potentially help understand the GP-340-bacteria binding mechanism.

Because of its good extraction efficiency and its importance in food safety the pathogen *L. monocytogenes* was chosen to be tested in co-culture with *P. paracarnis*, as it is a non-pathogenic and ubiquitous bacterium. Interestingly, magnetic extraction in the presence of *P. paracarnis* still appeared to be effective, as very low numbers of *Listeria* were detected in the supernatants. However, quantitative yields of *L. monocytogenes* in the magnetic particle fractions of the co-culture experiments were lower compared to those in pure inoculations. This may be explained by increased loss of *Listeria* during the washing steps due to competition with *Pseudomonas* for binding to the SPION^Pep^. In addition, altered growth behavior caused by an excess of *Pseudomonas* on the growth medium, as well as differences in nanoparticle aggregation resulting from higher bacterial numbers, may also have contributed to this effect.

It has not only been shown that there is a strong influence of the type of microorganism, e.g., gram type, but also matrix-associated properties. We observed lowered extraction efficiencies in the presence of meat matrix compared to pure inoculums for *L. monocytogenes*. Furthermore, extraction efficiencies of *Listeria* varied between different meat product types analyzed (7–31% for meat spread, 1–3% for minced meat). This could be attributed to different amounts of proteins, carbohydrates and fats in the matrices. It is known that the binding mechanism, though not fully understood, is based on the calcium dependent interaction between the binding motif VEVLXXXXW and poly-sulfated and poly-phosphated ligands (End et al. 2009; Purushotham & Deivanayagam 2014) but also leucine-rich repeats of bacterial surface proteins (Loimaranta et al. 2009). Different properties of food matrices could affect these binding mechanisms for example either by binding the peptide itself or by influencing the amount of needed calcium. These aspects can be studied in future endeavors.

The successful magnetic extraction can also be confirmed by confocal laser scanning microscopy. All visible bacteria (stained with FM4-64) observed during imaging were co-located to particle clusters indicating stable bonds between bacteria and nanoparticles (not shown). Exact nanoparticle forms and sizes could not be confirmed via CRM since signals from SPIONs primarily arise from optical reflections at particle cores or aggregates. Due to the microscope’s point-spread function (airy disk), even individual nanoparticles appeared strongly broadened, so the measured signal reflected the optical extent of the scattering object rather than its true physical or hydrodynamic size.

In conclusion it can be said that peptide functionalized SPIONs promise a reliable method of detecting pathogens in pure culture but also in food samples with the need for optimization of efficiencies of some pathogens. This diagnostic procedure should be further investigated and be coupled with subsequent identification tools, e.g., qPCR or critical offset magnetic particle spectroscopy (COMPASS, (Friedrich et al. 2023b)) for faster results.

## Supplementary Information

Below is the link to the electronic supplementary material.

ESM 1 (PDF 407 KB)

## Data Availability

The datasets used during the current study are available from the corresponding author upon reasonable request. All data generated or analyzed during this study are included in this published article and its supplementary information file.
