# Supplementary material for: Removal and analysis of foodborne-associated pathogens via peptide functionalized nanoparticles
Source: Appl Microbiol Biotechnol. 2026 Jul 6;110(1):203. doi: 10.1007/s00253-026-13922-x (PMC13337806; doi:10.1007/s00253-026-13922-x)
Supplement: Supplementary file 1 — (PDF 407 KB) [file 253_2026_13922_MOESM1_ESM.pdf]

Appendix (Supplementary information)

Removal and Analysis of Foodborne Associated Pathogens via Peptide Functionalized Nanoparticles

Emily Hausen[1], Sebastian Knorr[3], Stefan Lyer[1,2], Christoph Alexiou[1], Rainer Tietze[1]*, Sonja Lick[3]

1 Department of Otorhinolaryngology, Head and Neck Surgery, Section of Experimental Oncology and Nanomedicine (SEON), Else Kröner-Fresenius-Stiftung Professorship, Uniklinikum Erlangen, Germany

2 Department of Otorhinolaryngology, Head and Neck Surgery, Section of Experimental Oncology and Nanomedicine (SEON), Professorship for AI-assisted Nanomaterials, Uniklinikum Erlangen, Germany

3 Max Rubner-Institute (MRI), Department of Safety and Quality of Meat, Kulmbach, Germany

*Corresponding author: rainer.tietze@uk-erlangen.de

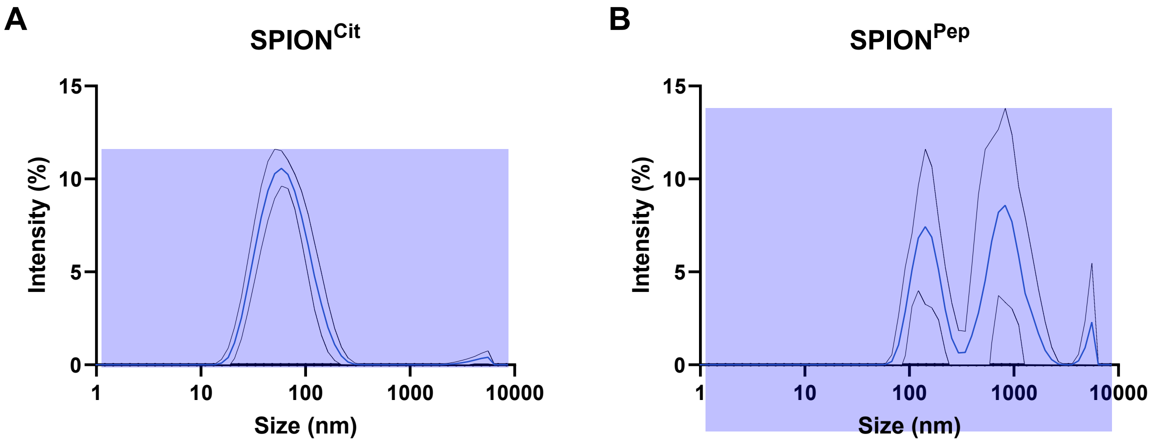

Fig. A 1 Intensity rated plots measured via Dynamic Light Scattering (DLS). A) Intensity profile of $SPION^{Cit}$ synthesized in an alkaline coprecipitation. B) Intensity profile of $SPION^{Pep}$. Shown is the growth of particles after functionalization with the peptide and due to the polydispersity, a non-reliable z-average.

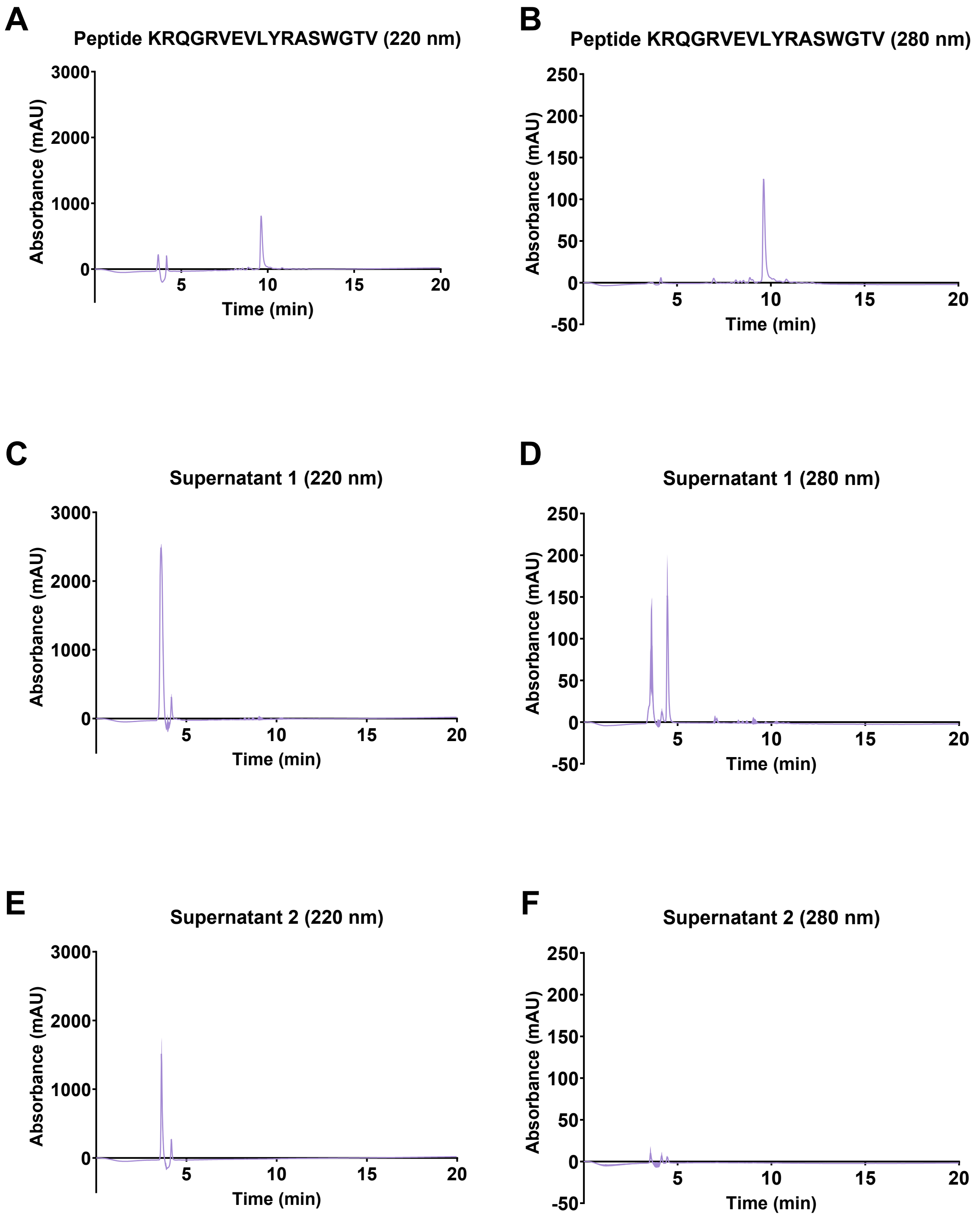

Fig. A 2 Chromatograms of used peptide KRQGRVEVLYRASWGTV and supernatants of the bindings. A) and B) show the chromatograms of the peptide KRQGRVEVLYRASWGTV dissolved in water and diluted to the concentration that was used for the binding. C) and D) show the chromatograms of the supernatants directly after binding; E) and F) the ones after washing the functionalized $SPION^{Pep}$. A), C) and E) show the chromatograms at the absorbance wavelength 220 nm, B), D) and F) at 280 nm.

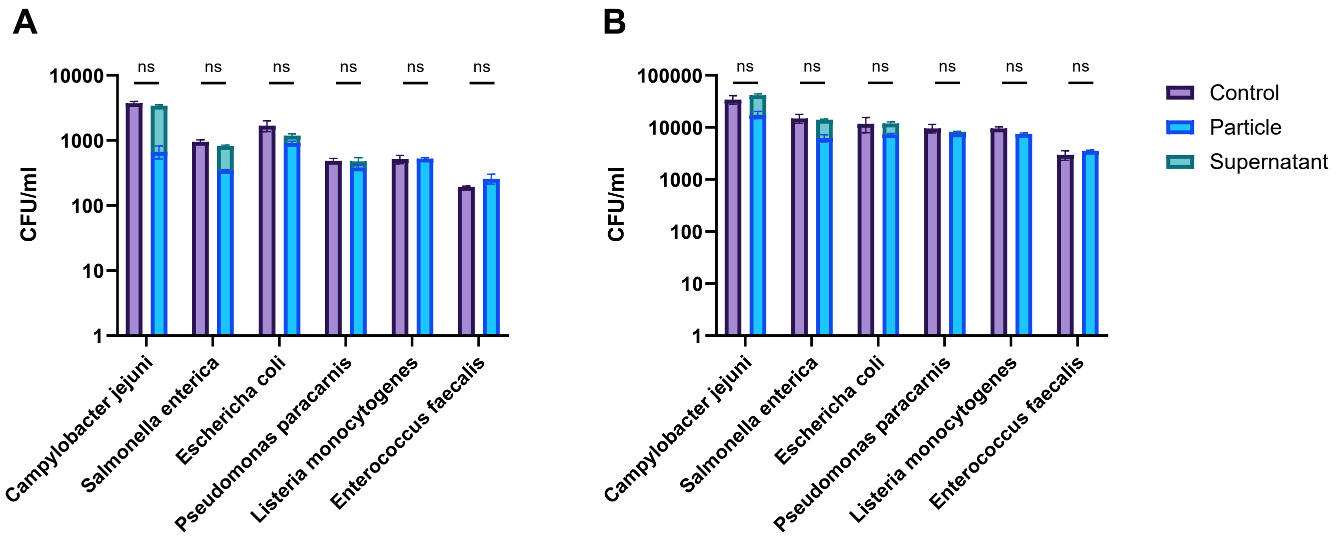

Fig. A 3 Validation of the magnetic extraction procedure. CFU/ml of the untreated control compared to the combined particle and supernatant fractions at 10^3 (A) and 10^4 CFU/ml (B) for all tested bacterial species. No significant differences were detected between fractions (Mann-Whitney U test with False Discovery Rate (FDR) correction (two-stage step-up method of Benjamini, Krieger and Yekutieli; all $q > 0.05$)

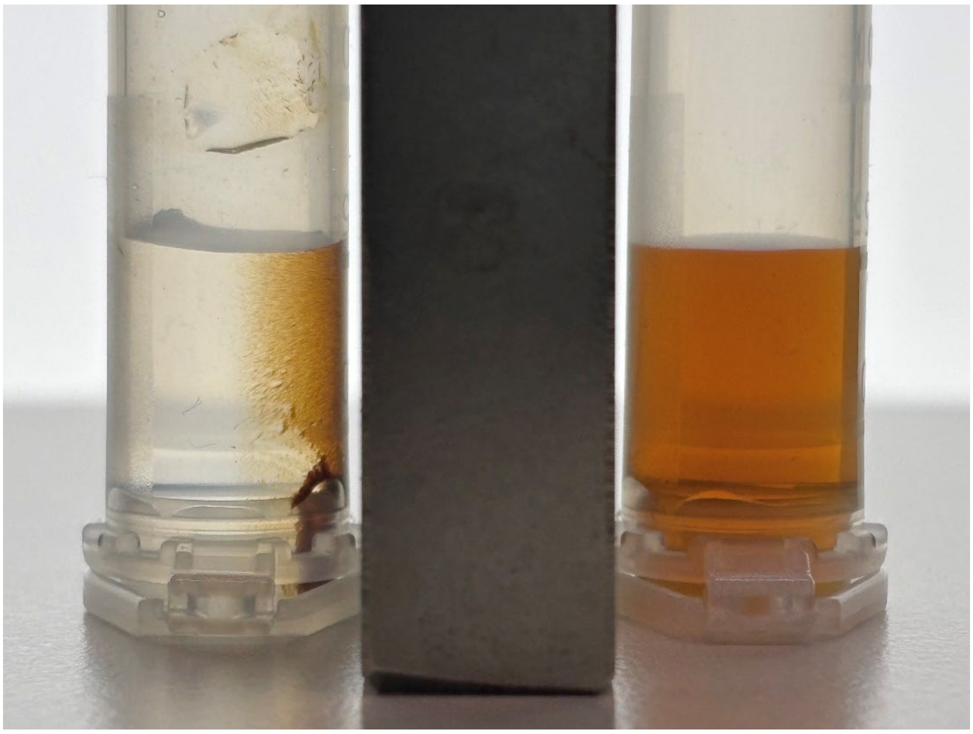

Figure A 4 Magnetic extraction of SPIONs in Ringer solution after three minutes (left tube: functionalized $SPION^{Pep}$, right tube: unmodified $SPION^{Cit}$). $SPION^{Cit}$ cannot be collected magnetophoretically, whereas $SPION^{Pep}$ shows an accumulation close to the magnet.

Table A 1 Raw CFU/ml data for bacterial extraction with SPION^{Pep} from pure culture at 10^3 and 10^4 CFU/ml. Tested pathogens were *Campylobacter jejuni, Salmonella enterica, Escherichia coli, Pseudomonas paracarnis, Listeria monocytogenes* and *Enterococcus faecalis*. Values represent mean ± SD of n = 3 independent replicates.

	**10^3 CFU/ml**			**10^4 CFU/ml**		
	Particle (CFU/ml)	Supernatant (CFU/ml)	Control (CFU/ml)	Particle (CFU/ml)	Supernatant (CFU/ml)	Control (CFU/ml)
Campylobacter jejuni	670.0 ± 151.7	2770.0 ± 105.8	3743.3 ± 217.3	8762.0 ± 1382.1	24087.7 ± 2393.0	34181.7 ± 6492.5
Salmonella enterica	348.3 ± 7.6	466.7 ± 32.1	953.3 ± 66.6	6218.3 ± 1003.6	7802.3 ± 384.5	14962.0 ± 2976.4
Escherichia coli	915.0 ± 60.6	270.0 ± 75.5	1686.7 ± 320.2	7421.0 ± 278.6	4519.0 ± 841.6	11708.0 ± 3813.5
Pseudomonas paracarnis	383.3 ± 36.9	93.3 ± 68.1	483.3 ± 46.2	7686.2 ± 800.1	530.0 ± 43.6	9596.0 ± 1749.4
Listeria monocytogenes	528.3 ± 16.1	0.0 ± 0.0	513.3 ± 76.4	7449.5 ± 358.6	6.7 ± 5.8	9568.3 ± 692.3
Enterococcus faecalis	258.3 ± 45.1	0.0 ± 0.0	193.3 ± 5.8	3567.0 ± 109.1	0.0 ± 0.0	2959.3 ± 610.8

Table A 2 Raw CFU/ml data for bacterial extraction of *L. monocytogenes* in co-culture (*Listeria monocytogenes* in surplus of *Pseudomonas paracarnis*) at 10, 100 and 1000 CFU/ml *Listeria monocytogenes*, respectively. Values represent mean ± SD of n = 3 independent replicates.

	Particle (CFU/ml)	Supernatant (CFU/ml)	Control (CFU/ml)
**10 CFU/ml**	4.3 ± 2.3	0.7 ± 0.6	10.3 ± 3.2
**100 CFU/ml**	35.3 ± 3.8	0.7 ± 0.6	102.0 ± 3.5
**1000 CFU/ml**	390.0 ± 37.7	5.3 ± 4.2	1253.0 ± 285.9

Table A 3 Raw CFU/ml data for bacterial extraction of *L. monocytogenes* in meat matrices (meat spread and minced meat) at 10, 100, 1000 and 10000 CFU/ml *Listeria monocytogenes*, respectively. Values represent mean ± SD of n = 3 independent replicates.

	**Meat Spread**			**Minced Meat**		
	Particle (CFU/ml)	Supernatant (CFU/ml)	Control (CFU/ml)	Particle (CFU/ml)	Supernatant (CFU/ml)	Control (CFU/ml)
**10 CFU/ml**	1.3 ± 0.0	12.0 ± 3.0	19.2 ± 3.4	0.3 ± 0.4	18.7 ± 3.1	19.7 ± 3.3
**100 CFU/ml**	48.3 ± 8.0	105.0 ± 27.8	208.3 ± 24.7	2.5 ± 2.5	250.0 ± 22.9	245.0 ± 13.2
**1000 CFU/ml**	571.7 ± 25.3	956.7 ± 75.1	1845.0 ± 116.5	65.8 ± 22.7	2211.7 ± 50.3	2338.3 ± 53.0
**10000 CFU/ml**	4835.8 ± 811.0	8272.5 ± 1294.5	13929.3 ± 690.9	373.3 ± 25.2	17579.2 ± 737.3	19329.3 ± 2511.8
